# Prognostic Value of the C-PLAN Index in Metastatic Renal Cell Carcinoma Treated with Nivolumab

**DOI:** 10.3390/jcm14072217

**Published:** 2025-03-25

**Authors:** Gökhan Şahin, Caner Acar, Haydar Çağatay Yüksel, Salih Tünbekici, Fatma Pınar Açar, Erhan Gökmen, Burçak Karaca

**Affiliations:** Department of Medical Oncology, Faculty of Medicine, Ege University, 35100 İzmir, Türkiye

**Keywords:** metastatic renal cell carcinoma, C-PLAN index, nivolumab, overall survival, progression-free survival, prognostic biomarker

## Abstract

**Background/Objectives**: Nivolumab has been shown to be an effective treatment for metastatic renal cell carcinoma (mRCC); however, patient responses vary considerably. The objective of this study is to evaluate the prognostic value of the C-PLAN index in predicting survival outcomes for patients with mRCC treated with nivolumab. **Methods**: This retrospective cohort study included 81 mRCC patients previously treated with tyrosine kinase inhibitors who subsequently received nivolumab. The C-PLAN index, which includes C-reactive protein, performance status, lactate dehydrogenase, albumin, and derived neutrophil-to-lymphocyte ratio, was used to classify patients into “good” and “poor” prognostic groups. **Results**: The median overall survival (OS) was 22 months, and the median progression-free survival (PFS) was 6.7 months. Patients in the “poor” C-PLAN group exhibited significantly shorter OS and PFS than those in the “good” group (median OS: 13 vs. 31 months, *p* = 0.003; median PFS: 3 vs. 10 months, *p* = 0.007). The C-PLAN index was identified as an independent predictor of both OS (HR = 1.19, 95% CI: 1.11–3.43, *p* = 0.020) and PFS (HR = 1.71, 95% CI: 1.04–2.78, *p* = 0.032) in multivariate analysis. **Conclusions**: These findings suggest that the C-PLAN index may serve as a valuable prognostic tool, offering insights into survival outcomes for patients undergoing nivolumab therapy. Further prospective and multicenter studies are warranted to validate its clinical utility.

## 1. Introduction

There are approximately 400,000 new cases of renal cell cancer (RCC) and over 170,000 deaths from RCC worldwide each year [1]. Approximately 50% of RCCs are found incidentally and 25% of patients are diagnosed at the metastatic stage, where prognosis remains poor despite advances in treatment strategies [2,3]. Patients with metastatic RCC (mRCC) have a high mortality risk [4]. Advanced RCC has high morbidity and mortality, and 5-year survival is less than 20% [5]. While adjuvant treatment options remain limited, there has been significant progress in the treatment of mRCC. Anti-vascular endothelial growth factor (VEGF) targeted tyrosine kinase inhibitors (TKIs) and immune checkpoint inhibitors (ICIs) have become the main treatment options due to their ability to significantly extend survival in patients with mRCC [6,7,8]. In recent years, combinations of ICIs and TKIs have been shown to improve survival in these patients [9,10].

Nivolumab is an IgG4 monoclonal antibody that targets the programmed death-1 (PD-1) receptor. The CheckMate 025 study comparing nivolumab with everolimus in mRCC patients previously treated with anti-VEGF TKIs showed superiority of nivolumab and has been used as monotherapy in advanced RCC patients who progressed after prior antiangiogenic therapy [8]. However, the clinical benefits of nivolumab treatment may not be the same across all mRCC patients. For example, 35% of patients did not derive any benefit from nivolumab in the CheckMate 025 trial. Given the cost of ICI treatment, it is important to identify predictive or prognostic markers that can help tailor therapy for mRCC patients. Biomarkers that predict clinical outcomes with nivolumab can help identify non-responders early and start alternative therapy on time.

The IMDC (International Metastatic RCC Database Consortium) score developed, during the anti-VEGF era, includes clinical parameters such as performance status, hemoglobin, calcium levels, and neutrophil and platelet counts to predict patient prognosis [11]. This scoring system classifies patients with mRCC into three distinct risk categories: favorable, intermediate, and poor. The IMDC score is a well-established prognostic tool for anti-VEGF therapy, providing reliable risk stratification in mRCC [12,13]. Although the IMDC score is a reliable prognostic marker for anti-VEGF therapy, its predictive power in immunotherapy response has been shown to be limited. The literature emphasizes the need for more specific prognostic markers for immunotherapy and highlights the limitations of the IMDC score due to its poor predictive capacity in immunotherapy treatment [14]. Inflammation is widely recognized as a key player in tumor development and response to anti-cancer therapies [15]. Studies have shown that systemic inflammation indicators, such as high C-reactive protein (CRP), neutrophils, and lymphocytes, may be effective in predicting the response to immunotherapy [16,17]. For example, a study by Roussel et al. (2021) showed that high CRP and neutrophil-to-lymphocyte ratio (NLR) were associated with poor outcomes in nivolumab-treated patients, highlighting the role of inflammation in treatment efficacy [18]. These findings suggest an increasing role of inflammation in immunotherapy efficacy and emphasize the need for developing more appropriate prognostic markers for clinical practice.

In addition, inflammatory markers, metabolic, and nutritional factors also play a crucial role in shaping immunotherapy outcomes. Elevated lactate dehydrogenase (LDH) can drive lactate production in the tumor microenvironment, potentially dampening immune checkpoint inhibitor (ICI) efficacy, whereas low serum albumin, indicative of poor nutritional status, correlates with worse survival in ICI-treated patients [19,20]. Thus, integrating inflammatory, metabolic, and nutritional parameters with clinical factors such as performance status may significantly enhance prognostic accuracy in immunotherapy-treated mRCC, ultimately guiding more effective treatment decisions.

The C-PLAN index, which consists of C-reactive protein, performance status, lactate dehydrogenase, albumin, and derived NLR, has been shown to be a prognostic factor for patients with previously untreated advanced non-small cell lung cancer undergoing combination immunotherapy [21]. Based on the data obtained in lung cancer, it is thought that the C-PLAN index may also have predictive value for immunotherapy in renal cell carcinoma. The aim of this study is to evaluate the prognostic utility of the C-PLAN index in patients with mRCC treated with nivolumab in the second line and beyond.

## 2. Materials and Methods

The medical and laboratory records of 94 consecutive patients with mRCC, who had previously received TKIs before initiating nivolumab treatment between January 2016 and August 2023 at our institution, were retrospectively analyzed. This study was approved by the Ege University Faculty of Medicine Ethics Committee (Approval Number: 24-11T/11, Approval Date: 14 November 2024) and was conducted in accordance with the Declaration of Helsinki. All data were anonymized. We excluded 13 patients due to missing laboratory values for the C-PLAN index, resulting in a final cohort of 81 patients.

The nivolumab dosage regimen was 3 mg/kg or 240 mg intravenously every 14 days, with individualized dose adjustments in patients experiencing immune-related adverse events (irAEs).

### 2.1. Statistical Analysis

Continuous variables are presented as medians with interquartile ranges and categorical variables as frequencies and percentages. Patient characteristics (e.g., sex, age, and ECOG PS, disease features (e.g., tumor type, pathology, metastasis sites, nephrectomy status, and sarcomatoid feature), treatment history and details, blood routine, and biochemical parameters within two weeks before nivolumab initiation were collected.

The cutoff date was 5 May 2024. In our analysis, Chi-square tests were used to compare categorical variables between good and poor C-PLAN groups. Univariate and multivariate Cox regression analyses were used to calculate 95% CI and HR to evaluate factors associated with OS and PFS. Kaplan–Meier analysis was used to estimate survival and log-rank test to compare survival curves between groups. *p* < 0.05 was considered statistically significant.

To further assess the predictive value of the C-PLAN index in nivolumab response, univariate logistic regression analysis was conducted for objective response rate (ORR), and both univariate and multivariate logistic regression analyses were performed for disease control rate (DCR). Variables with *p* < 0.1 in univariate analysis were included in the multivariate model to identify independent predictors of DCR. Results were reported as hazard ratios (HRs) with 95% confidence intervals (CIs).

### 2.2. C-PLAN Index Calculation

C-PLAN index was calculated by combining five factors: C-reactive protein (CRP), performance status (PS), lactate dehydrogenase (LDH), albumin (Alb), and derived neutrophil-to-lymphocyte ratio (dNLR). Laboratory values used to calculate the C-PLAN index were collected within two weeks before nivolumab treatment. Cutoff values and scoring criteria for these factors are summarized in Table 1. CRP levels < 1.0 mg/dL were scored as 0, and ≥1.0 mg/dL as 1. Similarly, PS was scored as 0 for patients with a performance status of 0–1 and as 1 for PS ≥ 2. LDH levels < 223 U/L, Alb ≥ 3.5 g/dL, and dNLR < 3 were each scored as 0, whereas LDH ≥ 223 U/L, Alb < 3.5 g/dL, and dNLR ≥ 3 were each scored as 1 (Table 1). C-PLAN index scores ranged from 0 to 5, with a median of 2. Patients were categorized into two groups: scores 0–1 were defined as ‘good’, and scores 2–5 as ‘poor’ [11]. Patients were divided into two groups accordingly.

Baseline characteristics, including age, sex, metastasis status, nephrectomy status, histological type, sarcomatoid feature, metastasis sites, ECOG status, nivolumab line, and IMDC risk category, were recorded and compared between the two groups.

### 2.3. Outcome Definitions

Treatment efficacy was evaluated using the Response Evaluation Criteria in Solid Tumors (RECIST 1.1), with radiologic assessments performed at regular intervals [22]. Additionally, clinicians’ assessments, including radiologic findings and documentation of clinical progression, were integrated into the response evaluation. Objective response rate (ORR) was defined as the proportion of patients achieving a complete response (CR) or partial response (PR) to nivolumab treatment. CR was defined as the disappearance of all target lesions, while PR was defined as at least a 30% decrease in the sum of the diameters of target lesions, as per RECIST 1.1 criteria. Disease control rate (DCR) was defined as the proportion of patients achieving CR, PR, or stable disease (SD). To assess the independent association between the C-PLAN index and treatment response, ORR and DCR were analyzed using logistic regression models.

Progression-free survival (PFS) was defined as the time from nivolumab initiation to disease progression or death from any cause. Overall survival (OS) was defined as the time from nivolumab initiation to death from any cause.

## 3. Results

### 3.1. Patient Characteristics in the C-PLAN Good and Poor Groups

The median age of all patients was 62 years (IQR: 54.5–70.0), with a slight difference between the groups (59 years for the good C-PLAN index group and 65 years for the poor C-PLAN index group, *p* = 0.302). Among the 81 patients, the distribution of first-line TKI therapies was as follows: pazopanib (51.9%), sunitinib (43.2%), and cabozantinib (4.9%). The distribution of these agents between the C-PLAN good and poor groups was not significantly different (*p* = 0.350). There were no statistically significant differences between groups in terms of gender, histological type, presence of sarcomatoid features, or metastatic sites (all *p* > 0.05). However, a significant difference was found for ECOG performance status, with the poor C-PLAN group having more patients with ECOG ≥ 2 compared to the good C-PLAN group (*p* = 0.01). IMDC risk classification also differed significantly between the groups (*p* < 0.001), with a higher proportion of patients in the poor risk category in the poor C-PLAN group (Table 2).

### 3.2. Survival Analiyses

**Figure 1:**
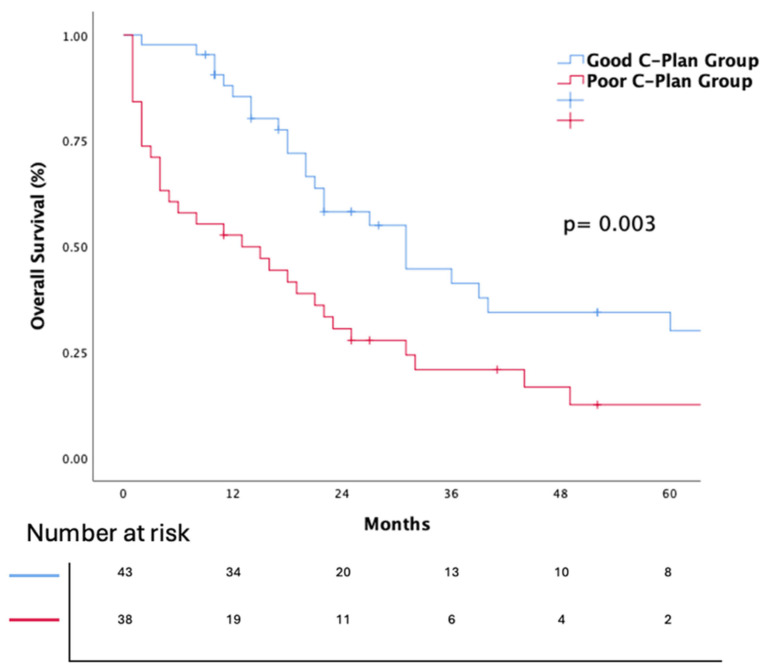
Kaplan–Meier estimates of overall survival according to C-plan groups (*p* value was calculated using the log-rank test).

**Figure 2:**
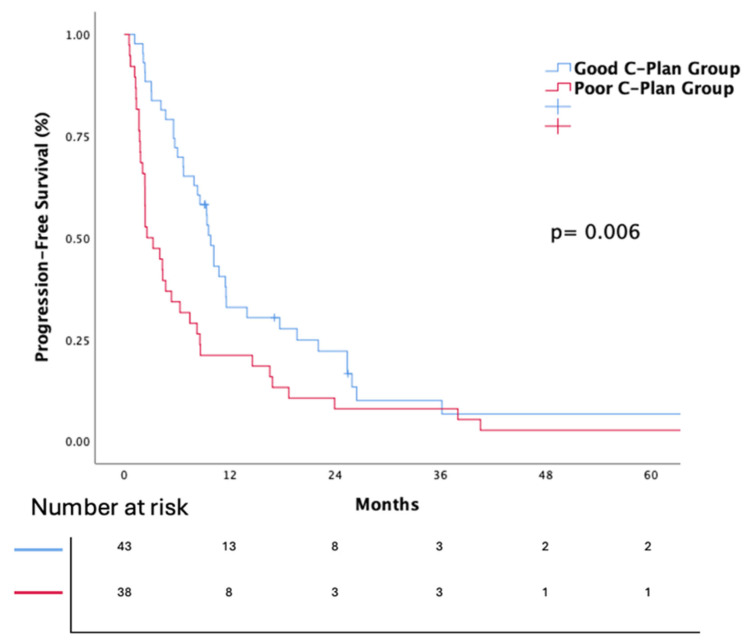
Kaplan–Meier estimates of progression-free survival according to C-plan groups (*p* value was calculated using the log-rank test).

The Kaplan–Meier survival analyses for PFS and OS for the entire cohort are presented in Figure 1 and Figure 2. The median OS was 22 months (95% Confidence Interval (CI): 17.9–26). The median OS was significantly different between the groups, with the good C-PLAN group showing a median OS of 31 months (95% CI: 19.8–42.1), compared to 13 months (95% CI: 1.3–24.6) in the poor C-PLAN group. The log-rank test confirmed the statistical significance of this difference (*p* = 0.003), indicating that patients with a good C-PLAN index had significantly better survival outcomes. The median PFS was 6.7 months (95% CI: 4.1–9.4). The median PFS was 10 months (95% CI: 8.2–11.7) in the good C-PLAN group and 3 months (95% CI: 1.8–4.1) in the poor C-PLAN group. The difference between the good and poor C-PLAN groups was statistically significant (HR: 1.87, 95% CI: 1.2–2.9, *p* = 0.007).

In univariate analysis for OS, an ECOG performance status of ≥2 was associated with a significantly increased mortality risk (HR = 2.17, 95% CI: 1.22–3.86, *p* = 0.008); however, this association lost statistical significance in the multivariate model (HR = 1.49, 95% CI: 0.79–2.83, *p* = 0.214). Similarly, IMDC risk classification showed a trend toward significance in univariate analysis (HR = 2.08, 95% CI: 0.86–5.00, *p* = 0.067) but did not retain independent prognostic value in the multivariate analysis (HR = 1.05, 95% CI: 0.37–2.97, *p* = 0.994). Brain metastasis (BM) was strongly associated with an increased mortality risk in both univariate (HR = 8.770, 95% CI: 2.79–27.51, *p* < 0.001) and multivariate analysis (HR = 6.71, 95% CI: 2.06–21.89, *p* = 0.002), emphasizing its substantial impact on OS. The poor C-PLAN index group had a significantly higher mortality risk in univariate analysis (HR = 2.162, 95% CI: 1.27–3.67, *p* = 0.005). This association remained statistically significant in the multivariate model (HR = 1.195, 95% CI: 1.11–3.43, *p* = 0.020), reinforcing its independent prognostic value in mRCC patients treated with nivolumab (Table 3).

For PFS, univariate analysis showed that a poor C-PLAN index was significantly associated with a higher risk of disease progression (HR = 1.87, 95% CI: 1.18–2.96, *p* = 0.008). ECOG performance status 2–4 was also associated with higher risk (HR 1.68, 95% CI 1.01–2.80, *p* = 0.047). BM and liver metastases (LM) were also significantly associated with progression risk, with HRs of 5.89 (95% CI: 1.96–17.72, *p* = 0.002) and 1.84 (95% CI: 1.09–3.11, *p* = 0.021), respectively. In the multivariate model, the C-PLAN index, BM, and LM remained significantly associated with PFS. Patients in the poor C-PLAN group had an HR of 1.71 (95% CI: 1.04–2.78, *p* = 0.032), those with BM had an HR of 4.08 (95% CI: 1.55–15.03, *p* = 0.007), and patients with LM had an HR of 2.03 (95% CI: 1.17–3.52, *p* = 0.012) (Table 4).

### 3.3. Efficacy of Nivolumab

Nivolumab efficacy in good and poor C-PLAN index groups is shown in Table 5. ORR (95% CI) for all patients, good C-PLAN index and poor C-PLAN index were 22.8%, 31.0% and 13.5%, respectively. The ORR showed no significant difference between the good and poor C-PLAN index groups (*p* = 0.065), although there was a trend towards significance. DCR (95% CI) for all patients, good C-PLAN index and poor C-PLAN index were 50.6%, 66.7%, and 32.4%, respectively. Good C-PLAN index group had significantly higher DCR than poor C-PLAN index group (*p* = 0.002).

To further evaluate the independent predictive value of the C-PLAN index on nivolumab efficacy, we performed univariate logistic regression analysis for ORR and both univariate and multivariate logistic regression analyses for DCR. For ORR, univariate analysis showed a trend towards significance (HR: 0.35, 95% CI: 0.11–1.01, *p* = 0.072), but due to the lack of statistical significance, multivariate analysis was not conducted (Table S1). For DCR, the C-PLAN index remained a significant independent predictor in multivariate analysis (HR: 0.33, 95% CI: 0.12–0.93, *p* = 0.035) (Table S2). These findings suggest that the C-PLAN index may be a useful prognostic tool in assessing nivolumab efficacy, particularly in predicting disease control.

## 4. Discussion

Nivolumab has shown great benefit in mRCC, but response varies widely among patients. Some have limited responses to treatment [23]. So, identifying predictive factors for nivolumab response is crucial for patient selection and better outcomes. In this regard, inflammation and nutritional status have been shown to be key in predicting response, and clinicians have valuable prognostic information. This study evaluates the prognostic value of the C-PLAN index in mRCC patients receiving nivolumab treatment. Our results show a higher C-PLAN index is associated with worse OS and PFS. To our knowledge, this is the first to show the C-PLAN index as a prognostic biomarker for nivolumab outcome in mRCC.

Systemic inflammation plays a pivotal role in tumor progression, immune modulation, and, ultimately, therapeutic efficacy, including ICIs. Recent studies have emphasized the prognostic significance of these inflammatory markers, especially in RCC treated with ICIs [24]. Elevated baseline levels of CRP and NLR correlate with poorer OS and PFS in metastatic clear–cell RCC patients treated with nivolumab [18]. Similarly, Mullally et al. reported that high dNLR is linked to worse survival outcomes in RCC patients receiving ICI therapy, highlighting systemic inflammation as a critical determinant of treatment response [25]. The Pan-Immune-Inflammation Value (PIV), which combines neutrophil, monocyte, platelet, and lymphocyte counts, has been shown to be an independent predictor of OS and PFS in patients on nivolumab beyond the first line [26]. Despite these findings, studies have used varying cut-off values for these biomarkers, which can limit the consistency and reliability of predictions. Many studies have also shown that individual inflammatory markers do not reliably predict survival on their own, which highlights the need for more integrated prognostic indices that combine these markers to improve predictive accuracy [27,28].

LDH is an enzyme that converts pyruvate to lactate and plays a key role in cellular metabolism. Elevated LDH levels are linked to increased lactate production, contributing to an immunosuppressive tumor microenvironment, which ultimately promotes tumor growth by altering the host immune response [29]. Moreover, inhibiting LDH in tumor cells has been shown to improve anti-PD-1 therapy [19]. Similarly, nutritional status is also a key factor in cancer treatment outcomes, and serum albumin is a marker of nutritional state and overall health. Low albumin levels have been associated with poor prognosis in patients undergoing ICI therapy [30]. Recent meta-analyses have demonstrated a significant association between hypoalbuminemia and worse survival outcomes, highlighting the prognostic importance of albumin in patients receiving ICIs [20]. In light of these findings, the Lung Immune Prognostic Index (LIPI), which combines LDH and derived neutrophil-to-lymphocyte ratio (dNLR), and the modified Glasgow Prognostic Score (mGPS), which incorporates serum albumin and C-reactive protein (CRP), were developed as prognostic tools [31,32] and have been investigated as biomarkers in mRCC patients treated with nivolumab [33,34].

The IMDC criteria, developed in the TKI era, remain the primary clinical tool for guiding treatment decisions in mRCC. However, they also have limitations, especially concerning immunotherapy outcomes [14]. This highlights the need for new, more reliable prognostic models. To address this gap, we evaluated the C-PLAN index. The C-PLAN index combines elements from both mGPS and LIPI, along with performance status (PS), to provide a comprehensive assessment of systemic inflammation, nutritional status, and overall patient performance [21].

In our study, both PFS and OS were significantly associated with the C-PLAN index in mRCC patients treated with nivolumab. In the multivariate analysis, the C-PLAN index remained an independent predictor for both OS and PFS, whereas ECOG and IMDC did not retain their predictive power. These findings suggest that the C-PLAN index may offer a more comprehensive prognostic value than the IMDC risk score for mRCC patients receiving nivolumab in the second-line setting and beyond. This supports the potential of the C-PLAN index as a reliable and integrative prognostic tool, encompassing markers of inflammation, nutritional status, and performance metrics.

The findings of our study highlight the significant predictive value of the C-PLAN index in determining nivolumab efficacy in mRCC patients. While DCR was significantly associated with the C-PLAN index, ORR did not reach statistical significance, though it demonstrated a trend toward significance. This lack of significance in ORR may be attributed to the limited sample size, which could have reduced statistical power. A larger cohort study may provide a more definitive conclusion regarding the association between the C-PLAN index and ORR.

The presence of BM was significantly associated with worse survival, with an increased mortality risk in both univariate and multivariate analyses in our study. While some studies, such as Hurwitz et al., support this finding by demonstrating shorter OS in mRCC patients with BM [35], others, including the NIVOREN trial and the Italian expanded access program, reported no significant difference in OS between BM and non-BM patients receiving nivolumab [36,37]. These inconsistencies may stem from differences in patient selection, prior therapies, or the biological characteristics of BM. Therefore, the prognostic role of BM in mRCC remains uncertain, underlining the need for further prospective studies to refine outcomes and improve treatment strategies for this subgroup.

LMs have been recognized as a negative prognostic factor in mRCC [38,39]. This trend has also been observed in mRCC patients receiving nivolumab, where LMs have been linked to poorer clinical outcomes [26,40]. In a large retrospective cohort study evaluating the impact of metastatic sites on ICI efficacy in genitourinary cancers, the presence of LM was significantly associated with worse PFS and OS [41]. In line with these reports, our study also found that LMs were associated with worse clinical outcomes, with a statistically significant impact on PFS. The association between LM and poorer survival may be explained by multiple factors, including an immunosuppressive tumor microenvironment, dysregulated T-cell function, and a more heterogeneous tumor composition, all of which can limit effective antitumor immunity [42,43].

In light of our findings on the predictive value of the C-PLAN index and the prognostic impact of both BM and LM, future multicenter and prospective studies with larger and more diverse patient cohorts are warranted to confirm and refine these results. Expanding the sample size and incorporating prospective analyses could help determine whether the C-PLAN index consistently predicts treatment response across different clinical settings. Additionally, a comparative evaluation of the C-PLAN index and the IMDC model within distinct risk categories may provide further insights into their relative prognostic utility. Investigating whether the C-PLAN index offers added predictive value beyond traditional IMDC scoring could refine patient stratification and optimize treatment decision-making in mRCC.

This study has several limitations. Firstly, the sample size was relatively small, and the limited number of female patients (n = 19) may restrict the generalizability of the findings across genders. Additionally, as a single-center and retrospective study, there is a risk of biases related to patient selection and data collection. Due to the retrospective design, PFS could not be consistently evaluated with predefined imaging intervals, potentially affecting the precision of PFS assessment. Furthermore, patients with missing data on key variables were excluded, which could impact the robustness of the findings and the interpretation of the C-PLAN index’s prognostic value. The laboratory values used to calculate the C-PLAN index may also have been influenced by external factors, such as infections or steroid use, potentially confounding the results. These limitations highlight the need for larger, prospective, multicenter studies to validate the utility of the C-PLAN index in mRCC and to establish its broader clinical applicability.

## 5. Conclusions

Our findings suggest that the C-PLAN index could serve as a practical and accessible prognostic tool for patients with mRCC undergoing nivolumab treatment in the second line and beyond. By incorporating markers of inflammation, nutritional status, and performance metrics, it has the potential to guide treatment decisions effectively. However, additional prospective and multicenter studies are needed to further validate its role in routine clinical practice.

## Figures and Tables

**Table 1:** C-PLAN Index Score.

Category	Score 0	Score 1
CRP (mg/dL)	<1.0	≥1.0
PS	0–1	≥2
LDH (U/L)	<223	≥223
Alb (g/dL)	≥3.5	<3.5
Derived NLR	<3.0	≥3.0

**Table 2:** Baseline characteristics.

Characteristics	All Patients (n = 81) (%)	c-Plan Good (n = 43) (%)	C-Plan Poor (n = 38) (%)	*p* Value
Age-years, median (IQR)	62 (54.5–70.0)	59 (53–70)	65(57–70)	0.302
Age group				0.169
<65	47 (58)	28 (65.1)	19 ((50)	
≥65	34(42)	15 (34.9)	19 (50)	
Gender				0.964
Male	62 (76.5)	33(76.7)	29 (76.3)	
Female	19 (23.5)	10(23.3)	9 (23.7)	
Metastase Status				0.480
Denovo	50 (61.7)	25 (58.1)	25 (65.8)	
Recurrence	31 (38.3)	18 (41.9)	13 (34.2)	
Previous nephrectomy				0.180
Yes	59 (72.8))	34 (79.1)	25 (65.8)	
No	22 (27.2)	9 (20.9)	13 (34.2)	
Histological type				0.859
Clear cell	61(75.3)	33 (76.7)	28 (73.7)	
Non-clear cell	11(13.6)	5 (11.6)	6 (15.8)	
Missing	9 (11.1)	5 (11.6)	4 (10.5)	
Sarcomatoid feature				0.488
Yes	7 (8.6)	4 (9.3)	3 (7.9)	
No	63 (77.8)	35 (81.4)	28 (73.7)	
Missing	11 (13.6)	4 (9.3)	7 (18.4)	
First-line TKI				0.350
Cabozantinib	4 (4.9)	1 (2.3)	3 (7.9)	
Pazopanib	42 (51.9)	21 (48.8)	21 (55.3)	
Sunitinib	35 (43.2)	21 (48.8)	14 (36.8)	
Metastatic sites				
Lung	52 (64.2)	27 (62.8)	25 (65.8)	0.779
Bone	42 (51.9)	25 (58.1)	17 (44.7)	0.228
Liver	20 (24.7)	8 (18.6)	12 (31.6)	0.177
Brain	4 (4.9)	0 (0)	4 (10.5)	**0.044**
ECOG				**0.010**
0–1	58 (71.6)	38 (88.4)	20 (52.6)	
≥2	21(25.9)	4 (9.3)	17 (44.7)	
Missing	2 (2.5)	1 (2.3)	1 (2.6)	
Nivolumab line				0.854
Second	52 (64.2)	28 (65.1)	24 (63.2)	
Third and beyond	29 (35.8)	15 (34.9)	14 (36.8)	
IMDC risk				**<0.001**
Favorable	10 (12.8)	6 (14)	4 (10.5)	
Intermediate	45 (57.7)	32 (74.4)	13 (34.2)	
Poor	23 (29.5)	4 (9.3)	19 (50)	
Missing	3 (14.5)	1 (2.3)	2 (5.3)	

Abbreviations: ECOG: Eastern Cooperative Oncology Group, IMDC: International Metastatic Renal Cell Carcinoma Database Consortium, IQR: Inter-quartile range, and TKI: Tyrosine Kinase Inhibitor.

**Table 3:** Univariate and multivariate Cox regression analysis for overall survival.

	Univariate		*p* Value ^a^	Multivariate		*p* Value ^a^
	HR	%95 CI		HR	%95 CI	
Age group			0.638			
<65	1					
≥65	1.13	(0.67–1.93)				
Gender			0.730			
Male	1.11	(0.60–2.07)				
Female	1					
Metastase Status			0.697			
Denovo	1.11	(0.65–1.91)				
Recurrence	1					
Previous nephrectomy			0.105			
Yes	1					
No	1.62	(0.90–2.92)				
Histological type			0.448			
Clear cell	1.39	(0.59–3.3)				
Non-clear cell	1					
Sarcomatoid feature			0.469			
Yes	1.37	(0.583–3.23)				
No	1					
ECOG			**0.008**			0.214
0–1	1			1		
≥2	2.17	(1.22–3.86)		1.49	(0.79–2.83)	
Nivolumab line			0.659			
Second	1					
Third and beyond	1.13	(0.65–1.94)				
IMDC risk			**0.067**			0.994
Favorable	1			1		
Intermediate	1.07	(0.46–2.46)		1.02	(0.44–2.36)	
Poor	2.08	(0.86–5.00)		1.05	(0.37–2.97)	
Liver Met			0.178			
Yes	1.49	(0.83–2.68)				
No	1					
Bone Met			0.726			
Yes	1.1	(0.64–1.86)				
No	1					
Lung Met			0.963			
Yes	1.01	(0.58–1.75)				
No	1					
Brain Met			**<0.001**			**0.002**
Yes	8.770	(2.79–27.51)		6.71	(2.06–21.89)	
No	1			1		
C-Plan			**0.004**			**0.020**
Good	1			1		
Poor	2.162	(1.27–3.67)		1.19	(1.11–3.43)	

ᵃ: *p*-values were calculated using Cox’s proportional hazards regression model.

**Table 4:** Univariate and multivariate analysis for progression-free survival.

	Univariate		*p* Value ^a^	Multivariate		*p* Value ^a^
	HR	%95 CI		HR	%95 CI	
Age group			0.472			
<65	1.18	(0.74–1.89)				
≥65	1					
Gender			0.675			
Male	1					
Female	1.12	(0.65–1.91)				
Metastase Status			0.883			
Denovo	1.04	(0.65–1.65)				
Recurrence	1					
Previous nephrectomy			0.425			
Yes	1					
No	1.24	(0.73–2.12)				
Histological type			0.837			
Clear cell	1					
Non-clear cell	1.07	(0.54–2.11)				
Sarcomatoid feature			0.289			
Yes	1					
No	1.58	(0.67–3.71)				
ECOG			**0.047**			0.271
0–1	1			1		
≥2	1.68	(1.01–2.80)		1.38	(0.77–2.47)	
Nivolumab line			0.761			
Second	1					
Third and beyond	1.01	(0.66–1.73)				
IMDC risk			0.116			
Favorable	1					
Intermediate	1.73	(0.74–3.50)				
Poor	2.29	(1.02–5.17)				
Liver Met			**0.021**			**0.012**
Yes	1.84	(1.09–3.11)		2.03	(1.17–3.52)	
No	1			1		
Bone Met			0.731			
Yes	1.08	(0.68–1.72)				
No	1					
Lung Met			0.873			
Yes	1					
No	1.04	(0.64–1.67)				
Brain Met						**0.007**
Yes	5.89	(1.96–17.72)	**0.002**	4.83	(1.55–15.03)	
No	1			1		
C-Plan			**0.008**			**0.032**
Good	1			1		
Poor	1.87	(1.18–2.96)		1.71	(1.04–2.78)	

ᵃ: *p*-values were calculated using Cox’s proportional hazards regression model.

**Table 5:** Efficacy of nivolumab therapy in the good and poor C-PLAN index groups.

Efficacy	All Patients, N (%)	Good C-PLAN Index Group, N (%)	Poor C-PLAN Index Group, N (%)	*p*-Value
CR	1 (1.2)	1 (2.3)	0 (0.0)	
PR	17 (21)	12(27.9)	5 (13.2)	
SD	22 (27.2)	15 (34.9)	7 (18.4)	
PD	39 (48.1)	14 (32.6)	25 (65.8)	
NE	2 (2.5)	1 (2.3)	1 (2.6)	
ORR, % (95%, CI)	22.8 (11.1–35.0)	31 (11.1–49.6)	13.5% (1.1–24.7)	0.065
DCR, % (95%, CI)	50.6 (40.0–61.2)	66.7(44.6–81.7)	32.4% (18.7–40.0)	**0.002**

Abbreviations: CI: confidence interval, CR: complete response, DCR: disease control rate, NE: Not evaluable, ORR: overall response rate, PD: progressive disease, PR: partial response, SD: stable disease.

## Data Availability

The datasets used and analyzed during the current study are not publicly available due to patient confidentiality but are available from the corresponding author upon reasonable request.

## Supplementary Materials

The following supporting information can be downloaded at: https://www.mdpi.com/article/10.3390/jcm14072217/s1, Table S1: Univariate Logistic Regression Analysis for Predictors of Objective Response Rate. Table S2: Univariate and Multivariate Logistic Regression Analysis for Predictors of Disease Control Rate.

## References

[1] World Health Organization (2023). Global Breast Cancer Initiative Implementation Framework: Assessing, Strengthening and Scaling-Up of Services for the Early Detection and Management of Breast Cancer.

[2] Patard J.-J. (2009). Incidental renal tumours. Curr. Opin. Urol..

[3] Lam J.S., Leppert J.T., Belldegrun A.S., Figlin R.A. (2005). Novel approaches in the therapy of metastatic renal cell carcinoma. World J. Urol..

[4] Mlcochova H., Machackova T., Rabien A., Radova L., Fabian P., Iliev R., Slaba K., Poprach A., Kilic E., Stanik M. (2016). Epithelial-mesenchymal transition-associated microRNA/mRNA signature is linked to metastasis and prognosis in clear-cell renal cell carcinoma. Sci. Rep..

[5] Bhat S. (2010). Role of surgery in advanced/metastatic renal cell carcinoma. Indian J. Urol..

[6] Motzer R.J., Rini B.I., Bukowski R.M., Curti B.D., George D.J., Hudes G.R., Redman B.G., Margolin K.A., Merchan J.R., Wilding G. (2006). Sunitinib in patients with metastatic renal cell carcinoma. JAMA.

[7] Motzer R.J., Hutson T.E., Cella D., Reeves J., Hawkins R., Guo J., Nathan P., Staehler M., de Souza P., Merchan J.R. (2013). Pazopanib versus sunitinib in metastatic renal-cell carcinoma. N. Engl. J. Med..

[8] Motzer R.J., Escudier B., McDermott D.F., George S., Hammers H.J., Srinivas S., Tykodi S.S., Sosman J.A., Procopio G., Plimack E.R. (2015). Nivolumab versus everolimus in advanced renal-cell carcinoma. N. Engl. J. Med..

[9] Choueiri T.K., Powles T., Burotto M., Escudier B., Bourlon M.T., Zurawski B., Oyervides Juárez V.M., Hsieh J.J., Basso U., Shah A.Y. (2021). Nivolumab plus cabozantinib versus sunitinib for advanced renal-cell carcinoma. N. Engl. J. Med..

[10] Rini B.I., Plimack E.R., Stus V., Gafanov R., Hawkins R., Nosov D., Pouliot F., Alekseev B., Soulières D., Melichar B. (2019). Pembrolizumab plus axitinib versus sunitinib for advanced renal-cell carcinoma. N. Engl. J. Med..

[11] Ko J.J., Xie W., Kroeger N., Lee J.-L., Rini B.I., Knox J.J., Bjarnason G.A., Srinivas S., Pal S.K., Yuasa T. (2015). The International Metastatic Renal Cell Carcinoma Database Consortium model as a prognostic tool in patients with metastatic renal cell carcinoma previously treated with first-line targeted therapy: A population-based study. Lancet Oncol..

[12] Dudani S., Savard M.-F., Heng D.Y. (2020). An update on predictive biomarkers in metastatic renal cell carcinoma. Eur. Urol. Focus.

[13] Heng D.Y., Xie W., Regan M.M., Harshman L.C., Bjarnason G.A., Vaishampayan U.N., Mackenzie M., Wood L., Donskov F., Tan M.-H. (2013). External validation and comparison with other models of the International Metastatic Renal-Cell Carcinoma Database Consortium prognostic model: A population-based study. Lancet Oncol..

[14] Rosellini M., Marchetti A., Mollica V., Rizzo A., Santoni M., Massari F. (2023). Prognostic and predictive biomarkers for immunotherapy in advanced renal cell carcinoma. Nat. Rev. Urol..

[15] Grivennikov S.I., Greten F.R., Karin M. (2010). Immunity, inflammation, and cancer. Cell.

[16] Iinuma K., Enomoto T., Kawada K., Fujimoto S., Ishida T., Takagi K., Nagai S., Ito H., Kawase M., Nakai C. (2021). Utility of neutrophil-to-lymphocyte ratio, platelet-to-lymphocyte ratio, and systemic immune inflammation index as prognostic, predictive biomarkers in patients with metastatic renal cell carcinoma treated with nivolumab and ipilimumab. J. Clin. Med..

[17] Yildirim H.C., Kus F., Guven D.C., Karaca E., Kaygusuz Y., Dizdar O., Aksoy S., Erman M., Yalcin S., Kilickap S. (2023). Mean Platelet Volume to Lymphocyte Ratio: A New Biomarker Predicting Response in Patients with Solid Tumors Treated with Nivolumab. J. Immunother. Precis. Oncol..

[18] Roussel E., Kinget L., Verbiest A., Debruyne P.R., Baldewijns M., Van Poppel H., Albersen M., Beuselinck B. (2021). C-reactive protein and neutrophil-lymphocyte ratio are prognostic in metastatic clear-cell renal cell carcinoma patients treated with nivolumab. Urol. Oncol. Semin. Orig. Investig..

[19] Daneshmandi S., Wegiel B., Seth P. (2019). Blockade of lactate dehydrogenase-A (LDH-A) improves efficacy of anti-programmed cell death-1 (PD-1) therapy in melanoma. Cancers.

[20] Guven D.C., Sahin T.K., Erul E., Rizzo A., Ricci A.D., Aksoy S., Yalcin S. (2022). The association between albumin levels and survival in patients treated with immune checkpoint inhibitors: A systematic review and meta-analysis. Front. Mol. Biosci..

[21] Sonehara K., Ozawa R., Hama M., Nozawa S., Agatsuma T., Nishie K., Kato A., Matsuo A., Araki T., Komatsu M. (2023). C-PLAN index as a prognostic factor for patients with previously untreated advanced non-small cell lung cancer who received combination immunotherapy: A multicenter retrospective study. Thorac. Cancer.

[22] Eisenhauer E.A., Therasse P., Bogaerts J., Schwartz L.H., Sargent D., Ford R., Dancey J., Arbuck S., Gwyther S., Mooney M. (2009). New response evaluation criteria in solid tumours: Revised RECIST guideline (version 1.1). Eur. J. Cancer.

[23] Fujiwara R., Inamura K., Yuasa T., Numao N., Yamamoto S., Masuda H., Kawauchi A., Takeuchi K., Yonese J. (2020). Efficacy and safety profile of nivolumab for Japanese patients with metastatic renal cell cancer. Int. J. Clin. Oncol..

[24] Rebuzzi S.E., Signori A., Banna G.L., Maruzzo M., De Giorgi U., Pedrazzoli P., Sbrana A., Zucali P.A., Masini C., Naglieri E. (2021). Inflammatory indices and clinical factors in metastatic renal cell carcinoma patients treated with nivolumab: The development of a novel prognostic score (Meet-URO 15 study). Ther. Adv. Med. Oncol..

[25] Mullally W.J., Greene J., Jordan E.J., Horgan A.M., O’Connor M., Calvert P.M. (2023). The prognostic value of the derived neutrophil-to-lymphocyte ratio (dNLR) in patients treated with immune checkpoint inhibitors. Ir. J. Med. Sci. (1971-).

[26] Yekedüz E., Tural D., Ertürk İ., Karakaya S., Erol C., Ercelep Ö., Arslan Ç., Sever Ö.N., Kılıçkap S., Şentürk Öztaş N. (2022). The relationship between pan-immune-inflammation value and survival outcomes in patients with metastatic renal cell carcinoma treated with nivolumab in the second line and beyond: A Turkish oncology group kidney cancer consortium (TKCC) study. J. Cancer Res. Clin. Oncol..

[27] Templeton A.J., McNamara M.G., Šeruga B., Vera-Badillo F.E., Aneja P., Ocaña A., Leibowitz-Amit R., Sonpavde G., Knox J.J., Tran B. (2014). Prognostic role of neutrophil-to-lymphocyte ratio in solid tumors: A systematic review and meta-analysis. J. Natl. Cancer Inst..

[28] Nishiyama N., Hirobe M., Kikushima T., Matsuki M., Takahashi A., Yanase M., Ichimatsu K., Egawa M., Hayashi N., Negishi T. (2020). The neutrophil-lymphocyte ratio has a role in predicting the effectiveness of nivolumab in Japanese patients with metastatic renal cell carcinoma: A multi-institutional retrospective study. BMC Urol..

[29] Romero-Garcia S., Moreno-Altamirano M.M.B., Prado-Garcia H., Sánchez-García F.J. (2016). Lactate contribution to the tumor microenvironment: Mechanisms, effects on immune cells and therapeutic relevance. Front. Immunol..

[30] Tang Q., Li X., Sun C.-R. (2024). Predictive value of serum albumin levels on cancer survival: A prospective cohort study. Front. Oncol..

[31] Mezquita L., Auclin E., Ferrara R., Charrier M., Remon J., Planchard D., Ponce S., Ares L.P., Leroy L., Audigier-Valette C. (2018). Association of the lung immune prognostic index with immune checkpoint inhibitor outcomes in patients with advanced non–small cell lung cancer. JAMA Oncol..

[32] Tsujino T., Komura K., Matsunaga T., Yoshikawa Y., Takai T., Uchimoto T., Saito K., Tanda N., Oide R., Minami K. (2017). Preoperative measurement of the modified glasgow prognostic score predicts patient survival in non-metastatic renal cell carcinoma prior to nephrectomy. Ann. Surg. Oncol..

[33] Carril-Ajuria L., Lavaud P., Dalban C., Negrier S., Gravis G., Motzer R.J., Chevreau C., Tannir N.M., Oudard S., McDermott D.F. (2024). Validation of the Lung Immune Prognostic Index (LIPI) as a prognostic biomarker in metastatic renal cell carcinoma. Eur. J. Cancer.

[34] Fujiwara R., Takemura K., Fujiwara M., Yuasa T., Yasuoka S., Komai Y., Numao N., Yamamoto S., Yonese J. (2021). Modified Glasgow prognostic score as a predictor of prognosis in metastatic renal cell carcinoma treated with nivolumab. Clin. Genitourin. Cancer.

[35] Hurwitz M.E., Considine B., Hasson N., Savion Gaiger N., Nelson M., Chiang V., Kluger H.M., Braun D.A., Schoenfeld D.A., Sznol M. (2025). Survival of patients with metastatic renal cell carcinoma with or without brain metastases. Am. Soc. Clin. Oncol..

[36] Escudier B.J., Chabaud S., Borchiellini D., Gravis G., Chevreau C., Brachet P.E., Geoffrois L., Laguerre B., Mahammedi H., Negrier S. (2017). Efficacy and safety of nivolumab in patients with metastatic renal cell carcinoma (mRCC) and brain metastases: Preliminary results from the GETUG-AFU 26 (Nivoren) study. Am. Soc. Clin. Oncol..

[37] Bracarda S., Galli L., Maruzzo M., Lo Re G., Buti S., Favaretto A., Di Costanzo F., Sacco C., Merlano M., Mucciarini C. (2018). Negative prognostic factors and resulting clinical outcome in patients with metastatic renal cell carcinoma included in the Italian nivolumab-expanded access program. Future Oncol..

[38] Dudani S., Guillermo de Velasco J., Gan C.L., Donskov F., Porta C., Fraccon A., Pasini F., Hansen A., Bjarnason G.A., Beuselinck B. (2020). Sites of Metastasis and Survival in Metastatic Renal-Cell Carcinoma (mRCC). Europe.

[39] Beuselinck B., Oudard S., Rixe O., Wolter P., Blesius A., Ayllon J., Elaidi R., Schöffski P., Barrascout E., Morel A. (2011). Negative impact of bone metastasis on outcome in clear-cell renal cell carcinoma treated with sunitinib. Ann. Oncol..

[40] Mollica V., Rizzo A., Tassinari E., Giunchi F., Schiavina R., Fiorentino M., Brunocilla E., Ardizzoni A., Massari F. (2021). Prognostic and predictive factors to nivolumab in patients with metastatic renal cell carcinoma: A single center study. Anti-Cancer Drugs.

[41] Ma V.T., Su C.T., Hu M., Taylor J.M., Daignault-Newton S., Kellezi O., Dahl M.N., Shah M.A., Erickson S., Lora J. (2021). Characterization of outcomes in patients with advanced genitourinary malignancies treated with immune checkpoint inhibitors. Urol. Oncol. Semin. Orig. Investig..

[42] Gerlinger M., Rowan A.J., Horswell S., Larkin J., Endesfelder D., Gronroos E., Martinez P., Matthews N., Stewart A., Tarpey P. (2012). Intratumor heterogeneity and branched evolution revealed by multiregion sequencing. N. Engl. J. Med..

[43] Limmer A., Ohl J., Kurts C., Ljunggren H.-G., Reiss Y., Groettrup M., Momburg F., Arnold B., Knolle P.A. (2000). Efficient presentation of exogenous antigen by liver endothelial cells to CD8+ T cells results in antigen-specific T-cell tolerance. Nat. Med..

