# Supplementary material for: Prognostic Value of the C-PLAN Index in Metastatic Renal Cell Carcinoma Treated with Nivolumab
Source: J Clin Med. 2025 Mar 25;14(7):2217. doi: 10.3390/jcm14072217 (PMC11989473; doi:10.3390/jcm14072217)
Supplement: Supplementary file 1 [file jcm-14-02217-s001.zip › jcm-3468727-supplementary.pdf]

Table S1. Univariate Logistic Regression Analysis for Predictors of Objective Response Rate

	Univariate		p value[a]
	HR	%95CI	
**Age group**			0.18
<65 vs ≥65	0.45	(0.14-1.43)	
**Gender**			**0.048**
Female vs Male	0.31	(0.1-0.99)	
**Metastase Status**			**0.064**
Recurrence vs Denovo	0.36	(0.12-1.06)	
**Previous nephrectomy**			**0.040**
Yes vs No	0.11	(0.14-0.90)	
**Histological type**			0.719
Clear cell vs Non-clear cell	1.31	(0.31-5.58)	
**Sarcomatoid feature**			0.818
No vs Yes	1.22	(0.22-6.70)	
**ECOG**			0.783
0-1 vs ≥2	0.84	(0.24-2.94)	
**Nivolumab line**			0.30
2nd vs $\geq 3^{rd}$	1.77	(0.60-5.19)	
**IMDC risk**			0.732
Favorable vs Intermediate	0.78	(0.17-3.54)	
Favorable vs Poor	0.52	(0.09-2.93)	
**Liver Met**			0.836
Yes vs No	0.88	(0.25-3.08)	
**Bone Met**			0.854
Yes vs No	0.91	(0.32-2.59)	
**Lung Met**			0.187
Yes vs No	0.49	(0.17-1.42)	
**C-Plan**			**0.072**
Good vs Poor	0.35	(0.11-1.01)	

Table S2. Univariate and Multivariate Logistic Regression Analysis for Predictors of Disease Control Rate

	Univariate		p value[a]	Multivariate		p value[a]
	HR	%95CI		HR	%95CI	
**Age group**			0.954			
<65 vs ≥65	0.97	(0.40-2.38)				
**Gender**			0.52			
Female vs Male	0.70	(0.24-2.08)				
**Metastase Status**			0.364			
Recurrence vs Denovo	0.65	(0.26-1.64)				
**Previous nephrectomy**			**0.090**			0.240
Yes vs No	0.41	(0.15-1.15)		0.53	(0.18-1.54)	
**Histological type**			0.873			
Clear cell vs Non-clear cell	0.90	(0.26-3.13)				
**Sarcomatoid feature**			0.10			
No vs Yes	6.2	(0.70-54.6)				
**ECOG**			**0.061**			0.240
0-1 vs ≥2	0.35	(0.18-1.05)		0.63	(1.18-2.13)	
**Nivolumab line**			0.99			
2nd vs $\geq 3^{rd}$	1.0	(0.39-2.53)				
**IMDC risk**			0.254			
Favorable vs Intermediate	0.88	(0.22-3.55)				
Favorable vs Poor	0.38	(0.08-1.77)				
**Liver Met**			0.137			
Yes vs No	0.45	(0.15-1.29)				
**Bone Met**			0.745			
Yes vs No	1.16	(0.48-2.80)				
**Lung Met**			0.364			
Yes vs No	0.65	(0.26-1.64)				
**C-Plan**			**0.006**			**0.035**
Good vs Poor	0.27	(0.11-0.69)		0.33	(0.12-0.93)	
